# Evaluation of lens dose from anterior electron beams: comparison of Pinnacle and Gafchromic EBT3 film

**DOI:** 10.1120/jacmp.v17i2.5713

**Published:** 2015-03-08

**Authors:** Marcus Sonier, Matt Wronski, Collins Yeboah

**Affiliations:** ^1^ Medical Physics, Sunnybrook Odette Cancer Centre Toronto ON Canada; ^2^ Department of Radiation Oncology University of Toronto Toronto ON Canada

**Keywords:** lens dose, lead shielding, electron beams, Hogstrom pencil beam algorithm, Pinnacle

## Abstract

Lens dose is a concern during the treatment of facial lesions with anterior electron beams. Lead shielding is routinely employed to reduce lens dose and minimize late complications. The purpose of this work is twofold: 1) to measure dose profiles under large‐area lead shielding at the lens depth for clinical electron energies via film dosimetry; and 2) to assess the accuracy of the Pinnacle treatment planning system in calculating doses under lead shields. First, to simulate the clinical geometry, EBT3 film and 4 cm wide lead shields were incorporated into a Solid Water phantom. With the lead shield inside the phantom, the film was positioned at a depth of 0.7 cm below the lead, while a variable thickness of solid water, simulating bolus, was placed on top. This geometry was reproduced in Pinnacle to calculate dose profiles using the pencil beam electron algorithm. The measured and calculated dose profiles were normalized to the central‐axis dose maximum in a homogeneous phantom with no lead shielding. The resulting measured profiles, functions of bolus thickness and incident electron energy, can be used to estimate the lens dose under various clinical scenarios. These profiles showed a minimum lead margin of 0.5 cm beyond the lens boundary is required to shield the lens to ≤10% of the dose maximum. Comparisons with Pinnacle showed a consistent overestimation of dose under the lead shield with discrepancies of ∼25% occurring near the shield edge. This discrepancy was found to increase with electron energy and bolus thickness and decrease with distance from the lead edge. Thus, the Pinnacle electron algorithm is not recommended for estimating lens dose in this situation. The film measurements, however, allow for a reasonable estimate of lens dose from electron beams and for clinicians to assess the lead margin required to reduce the lens dose to an acceptable level.

PACS number(s): 87.53.Bn, 87.53.Kn, 87.55.‐x, 87.55.D‐

## I. INTRODUCTION

Electron beams are an effective tool in the treatment of superficial lesions due to the plateau of the high‐dose region of the depth‐dose curve and steep dose falloff with depth. The penumbra of electron beams, however, broadens with depth due to increased multiple Coulomb scattering,^(1)^ leading to a bulging out of the low‐dose region. This bulging of the low isodose lines can pose a problem for radiosensitive organs at risk (OARs) directly adjacent to targets regardless of whether or not they are within the direct field. This is the case concerning irradiation of facial lesions and the proximity of the lens of the eye to the treatment field.

The lens of the eye is considered a highly radiosensitive organ with radiation‐induced cataractogenesis a main concern in modern radiotherapy. The incidence of cataract formation has been studied in the literature for single fraction and 2 Gy conventional fractionation schemes. Emami et al.^(2)^ estimated that there is a 5% and 50% risk of cataract formation at five years from 10 and 18 Gy considering dose incident on the lens of the eye. Alternate studies have proposed deterministic threshold doses of as low as 2 Gy acute doses and 4 Gy conventionally fractionated doses with further estimates that onset of cataract formation may occur after only 0.5 Gy suggesting a linear, no‐threshold complication model with a time to onset that is dose‐related.^3^, ^4^, ^5^, ^6^ As such, sparing of the lens during treatment is an important objective when preparing a patient's radiotherapy plan.

To achieve sufficient lens sparing, various techniques for the purposes of minimizing lens dose have been suggested depending on the site of the target. The first is the lens block used in the treatment of lesions within the eye such as: retinoblastoma and conjunctival lymphoma.^7^, ^8^, ^9^, ^10^ In this case, a pencil shield is positioned on the surface of the patient or with an offset, hanging in air from the electron applicator, with a thickness of 1 cm and a variable diameter ≥1.0 cm. The second is the internal eye shield designed to reduce the dose to the entire extent of the eye when treating the eyelid or canthus.^11^, ^12^, ^13^ Here, lead or tungsten hemispherical shields are placed directly onto the eye in treatments with low‐energy electrons (up to 9 MeV). The shields themselves are concentric with the eye with an anodized aluminum cap, dental acrylic, or wax layer on the proximal side of the shield to reduce electron backscatter dose to the interior of the eyelid. The third is large‐area skin collimation that is used to spare the eye and underlying structures of unnecessary dose when treating skin lesions adjacent to the eye such as those that may occur on the nose.^(11)^ Lead shielding is placed as skin collimation during treatment, covering the extent of the eye to maximally shield the lens with minimal effect on target coverage. When treating largely varying contours, superficial targets, or using higher energies, bolus may be placed atop the lead shielding and patient skin to create a flat surface for the electron beam to impinge upon, avoiding obliquity effects, and to achieve coverage of the target with the prescription isodose.

Determining the dose to the lens is not a simple task due to the cumbersome nature of *in vivo* dosimetry with the associated risk of inaccurate dosimeter placement, the difficulty in performing hand calculations for points distal to lead shielding, and the inaccuracy of modern treatment planning systems (TPSs) in the calculations of electron dose distributions with and without the presence of high density inhomogeneities.^11^, ^14^, ^15^ Thus, the purpose of this work is twofold: 1) to measure dose profiles under lead shielding at the lens depth for a range of clinical electron energies and bolus thicknesses enabling lens dose estimation in the clinic, and 2) to assess the validity of the Pinnacle TPS, currently in use at our center, in calculating the dose under lead shielding with the Hogstrom pencil beam electron algorithm. This study is specifically designed to address lens dose estimations for the situation of large‐area skin collimation with and without bolus.

## II. MATERIALS AND METHODS

### A. Radiochromic film measurements

First, Gafchromic EBT3 (Ashland Inc., Covington, KY) film calibration curves were created for incident electron energies of 6 and 18 MeV, the lowest and highest electron beam energies investigated, for the purpose of characterizing the film response as a function of electron energy.^(16)^ The dose‐response curve was a combination of the 6 and 18 MeV data and was generated for 0 to 40 Gy in the green channel due to the increased sensitivity observed at doses greater than 10 Gy and the improved accuracy over the range of doses deposited in the film from the high‐dose gradient at the lead edge compared to the red and blue channels.^(17)^ The Epson Expression 10000XL flatbed scanner (Epson America Inc., Long Beach, CA) was used to scan the irradiated films in a consistent orientation. All films were scanned in transmission mode with color corrections disabled, in 48‐bit color, and with a resolution of 150 dpi. Films used to construct the calibration curve were taken from the same batch as those to be used for measurements and left to stabilize for 24 hrs between irradiation and scanning.

**Figure 1:**
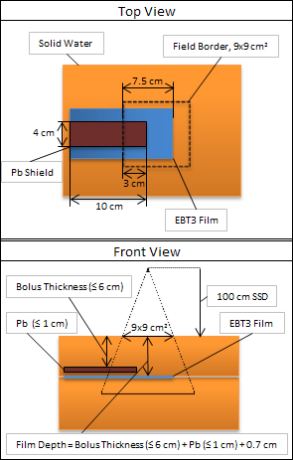
Film profile measurement phantom. The film is positioned at a depth of 0.7 cm beyond the bottom of the lead shielding with the edge aligned at 3 cm beyond the central axis of the incident beam. The lead shielding is aligned at 1.5 cm from the central axis of the beam (protruding 3 cm within the field border). The surface of the Solid Water phantom is positioned 100 cm SSD (not to scale).

Next, a phantom consisting of solid water and incorporating a lead shield of dimensions $4\times 10\;{\text{cm}}^{2}$ and a thickness dependent on the incident beam energy, as recommended in the AAPM Task Group 25 report, was constructed.^(18)^ Lead dimensions were selected such that the length of 10 cm extended beyond the field boundaries and the width of 4 cm removed any scattered dose contributions from points perpendicular to the off‐axis profile, centered at depth under the lead shield, for all energies investigated. Figure 1 illustrates the experimental setup. EBT3 film was positioned in the phantom such that 10 cm of solid water supplied sufficient backscatter with the film extending 3 cm beyond the central axis of the electron beam. A 0.7 cm thick piece of solid water was placed on top of the film to approximate the average depth of the lens within the eye. The lead shield was then positioned on top of the solid water such that it extended into the open field by 3 cm from the field border. To simulate bolus placement, a variable thickness of solid water was placed atop the lead shield with custom‐shaped solid water positioned laterally to the lead shield. A SSD of 100 cm and a field size of $9\times 9\;{\text{cm}}^{2}$ in a $10\times 10\;{\text{cm}}^{2}$ applicator were used for the irradiation. Off‐axis dose profiles extending from the open field beyond the central axis to the edge of the lead shield furthest from the central axis were then obtained. Table 1 displays the thicknesses of lead shielding and bolus used for each electron energy with the bolus thicknesses chosen to span the range of clinical scenarios used for treatment and allow for interpolation between the measured data. The monitor units (MUs) used for irradiation were selected to produce dose values in the lead‐shielded region at depth that were within the limits of the electron dose calibration curve. This resulted in selecting 2000 MUs for thinner bolus thicknesses and 4000 MUs for thicker bolus thicknesses where the film is located at an increased depth. The off‐axis dose profiles obtained from the film measurements were normalized to the central‐axis dose maximum in a homogeneous solid water phantom with no lead shielding. Identification of the edge of the lead shielding on the film was accomplished by projecting the divergence of the beam from the lead shield onto the film and noting the distance from the central axis of the beam. This then allowed for the distance from the edge of the film at 3 cm beyond the central axis to the lead shield to be determined.

**Table 1:** Thicknesses of lead shields and bolus employed for the measurements

*Energy (MeV)*	*Lead Thickness (cm)*	*Bolus Thicknesses (cm)*
6	0.4	0.5, 1, 1.5, 2
9	0.5	0.5, 1, 2, 3, 3.5
12	0.6	0, 1, 2, 3, 4.5
15	0.8	0, 1, 2.5, 4, 6
18	1.0	0, 1, 2.5, 4, 6

### B. Pinnacle model calculations

The geometry of the phantom in which the film measurements were performed was reproduced within our currently used clinical TPS, Pinnacle v9.2 (Philips Healthcare, Andover, MA). This was accomplished through the computed tomography (CT) reconstruction of a $30\times 30\times 30\;{\text{cm}}^{3}$ region set to a physical density of $1.0\;g\cdot {\text{cm}}^{-3}$ to produce a virtual phantom with a manual density override of $11.3\;g\cdot {\text{cm}}^{-3}$ applied at depth to simulate the presence of a lead shield producing the measurement phantom setup with applied bolus. Dose distributions were then generated using the Pinnacle pencil beam algorithm (PBA), with inhomogeneity corrections turned on and normalized to the dose at $d_{\text{max}}$ in a homogeneous phantom, for the selected energies and associated bolus thicknesses listed in Table 1.^15^, ^19^ The resultant off‐axis dose profiles, for a representative selection of electron energies and bolus thicknesses at a depth of 0.7 cm beyond the lead shielding and through the geometric centre of the electron field, were compared with the film measurements to assess the suitability of the heterogeneous PBA for lens dose calculations using lead shielding. Alignment of the film profile measurements and the Pinnacle dose profiles was achieved by projecting the beam divergence from the lead edge to a depth of 0.7 cm beneath the lead shielding and exporting data 5 cm in either direction along the dose profile such that the lead edge marked the center point.

## III. RESULTS

**Figure 2:**
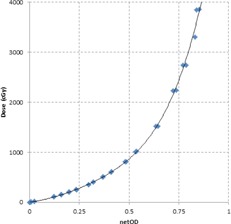
Electron film calibration curve (6‐18 MeV). The dose vs. net optical density calibration curve is a combination of data obtained for both 6 and 18 MeV electron energies with the resultant fit generated using all data points. Calculation of netOD follows the procedure of Sorriaux et al.^(20)^

**Figure 3:**
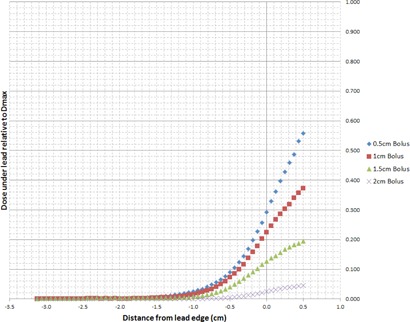
6 MeV EBT3 film profiles of relative dose vs. off‐axis position. Dose is relative to central axis dose maximum, $D_{\text{max}}$, in a homogeneous phantom with no lead shielding. The lead shielding extends from the inner edge (0 cm) to the outer edge (−10 cm); the inner edge is located at 1.5 cm from the central axis of the beam. Depth of measurement is 0.7 cm below the lead shielding of thickness 0.4 cm (depth=bolus thickness+0.4 cm+0.7 cm).

**Figure 4:**
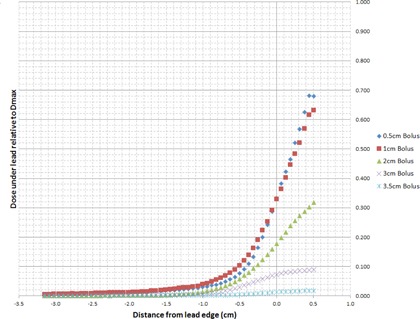
9 MeV EBT3 film profiles of relative dose vs. off‐axis position. Dose is relative to central axis dose maximum, $D_{\text{max}}$, in a homogeneous phantom with no lead shielding. The lead shielding extends from the inner edge (0 cm) to the outer edge (−10 cm); the inner edge is located at 1.5 cm from the central axis of the beam. Depth of measurement is 0.7 cm below the lead shielding of thickness 0.5 cm (depth=bolus thickness+0.5 cm+0.7 cm).

**Figure 5:**
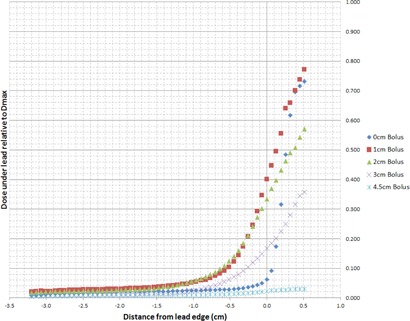
12 MeV EBT3 film profiles of relative dose vs. off‐axis position. Dose is relative to central axis dose maximum, $D_{\text{max}}$, in a homogeneous phantom with no lead shielding. The lead shielding extends from the inner edge (0 cm) to the outer edge (−10 cm); the inner edge is located at 1.5 cm from the central axis of the beam. Depth of measurement is 0.7 cm below the lead shielding of thickness 0.6 cm (depth=bolus thickness+0.6 cm+0.7 cm).

**Figure 6:**
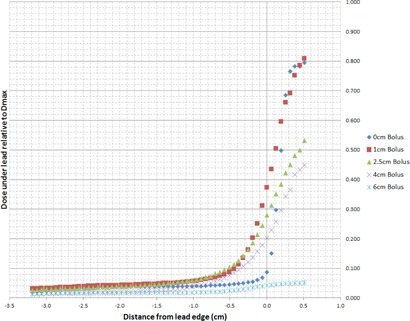
15 MeV EBT3 film profiles of relative dose vs. off‐axis position. Dose is relative to central axis dose maximum, $D_{\text{max}}$, in a homogeneous phantom with no lead shielding. The lead shielding extends from the inner edge (0 cm) to the outer edge (−10 cm); the inner edge is located at 1.5 cm from the central axis of the beam. Depth of measurement is 0.7 cm below the lead shielding of thickness 0.8 cm (depth=bolus thickness+0.8 cm+0.7 cm).

**Figure 7:**
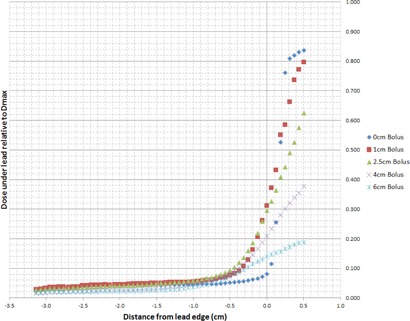
18 MeV EBT3 film profiles of relative dose vs. off‐axis position. Dose is relative to central axis dose maximum, $D_{\text{max}}$, in a homogeneous phantom with no lead shielding. The lead shielding extends from the inner edge (0 cm) to the outer edge (−10 cm); the inner edge is located at 1.5 cm from the central axis of the beam. Depth of measurement is 0.7 cm below the lead shielding of thickness 1.0 cm (depth=bolus thickness+0.1 cm+0.7 cm).

**Figure 8:**
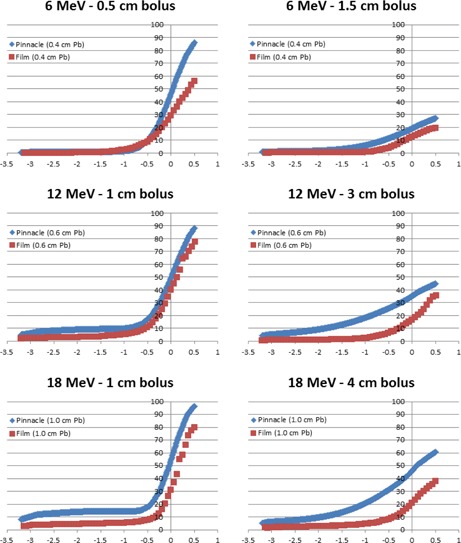
Pinnacle vs. EBT3 film dose profiles for anterior electron beam dose deposited under a lead shield. The 0 point on the x‐axis defines the edge of the lead shield with negative values denoting the region under the lead shield. The y‐axis reflects percent dose relative to $D_{\text{max}}$ with the profiles normalized to central‐axis dose maximum in a homogeneous phantom with no lead shielding.

The electron film calibration curves for 6 and 18 MeV were found to be energy independent and valid for use over the energy range investigated, consistent with studies in the literature.^(20)^ Figure 2 illustrates the electron calibration curve used to convert film net optical density in the green channel to dose for all energies under investigation. The measured off‐axis dose profiles extracted from the irradiated films for electron energies 6, 9, 12, 15, and 18 MeV are plotted in Figs. 3–7, which can serve the purpose of providing a simple lens dose lookup source for use in the clinic. Overlaid in each figure are the profile measurements at a given energy for all bolus thicknesses investigated. The zero position on the x‐axis marks the edge of the lead shield projected onto the film plane, accounting for beam divergence. Comparison plots between the Pinnacle TPS and film measurements for electron energies of 6, 12, and 18 MeV and selected bolus thicknesses listed in Table 1 are presented in Fig. 8.

## IV. DISCUSSION

There are two main effects that govern the dose profiles obtained via film dosimetry. The first of these effects is the skin collimation stopping the electrons in the incident beam due to the presence of the lead shielding. The second effect is due to the increased lateral scatter of the electron beam as the bolus thickness is increased. The increased width of the angular distribution of the electron beam at the level of the lead shielding with increasing bolus thickness results in a larger proportion of electrons deflected laterally, directly increasing the dose deposited at depth beneath the lead shielding.^(1)^ Thus, for each electron energy, as the bolus thickness increases, the dose gradient at the lead edge becomes less steep due to increased lateral scattering of the electrons at increased depths. The film profile results given in Figs. 3–7 display this effect for all clinical electron energies considered. Utilizing Figs. 3–7, the dose to the lens can be estimated by considering the choice of electron energy, the thickness of bolus proximal to the lead shield, and the distance of the lens from the lead edge.

In addition to estimating the dose to the lens using the data presented in this study, the clinician can determine the margin of lead shielding from the edge of the lens required to reduce the lens dose to an acceptable amount. This should enable the construction of lens shielding tailored to the clinical choice of electron energy and bolus thickness. The measured profiles indicate that, in most situations, a minimum margin of 0.5 cm of lead in one‐dimension from the edge of the lens with sufficient shielding on each of the other sides is necessary to reduce the lens dose to as low as 10% of the dose at $d_{\text{max}}$ in a homogeneous medium. Notably, these observations correlate with trends shown in a Monte Carlo study with similar measurement conditions that simulated dose profiles at a depth of 0.7 cm in a Solid Water phantom as a function of thickness and diameter of lead shielding placed on the phantom's surface and along the central axis for 4, 9, and 16 MeV electron beams.^(21)^ Illustrated in the figures of that study, across all energies, is the requirement of a margin of >0.5 cm in order for the lens to be shielded to <10% of the dose relative to $D_{\text{max}}$. These findings are identical to those obtained from the film dose profile measurements across the range of electron energies examined for the worst‐case scenario in which the bolus thickness is at a minimum. A margin of 0.5 cm thus represents our recommended minimum lateral extent of lead shielding that should be used. Effort should be made to maximize the extent of shielding while taking into account the choice of beam energy and patient‐specific considerations for each clinical scenario.

Comparisons between Pinnacle v9.2 calculations and the measured electron off‐axis dose profiles displayed in Fig. 8 reveals that the PBA consistently overestimates the dose deposited beneath the lead shielding. A contribution to this disagreement is the saturation of the electron stopping power lookup table as a function of physical density used in the Pinnacle TPS dose calculation. The current lookup table, shown in Table 2, extends to a maximum physical density of $7g\cdot {\text{cm}}^{-3}$, resulting in density overrides above this value to be assigned the same collision stopping power ratio and angular scattering power ratio as that for $7g\cdot {\text{cm}}^{-3}$. This results in the Pinnacle collision stopping power ratio to appear too low and allow some fraction of the incident electrons to penetrate the lead shield. This effect increases the dose deposited at depth and could be addressed by modifying the lead shield thicknesses to be greater than those used in patient collimation. In addition, the application of bolus upstream to high density skin collimation causes the central‐axis approximation of the PBA to break down resulting in highly inaccurate electron beam dose computations.^(22)^ Electrons scattered into the lead shield from the open field are not stopped in the PBA, incorrectly increasing dose deposited in the penumbral region under the lead shield while electrons incident over the lead shield and scattered into the open field are stopped in the PBA, incorrectly decreasing dose deposited in the penumbral region adjacent to the lead shielding. The net result of these contributing factors causes Pinnacle to calculate a penumbra that is too broad at the edge of the lead shield, depicted in the thicker bolus plots of Fig. 8 for each respective energy, with a smaller effect observed for thinner bolus and higher energy cases causing the penumbra to appear similar in width, but offset due to the electrons penetrating the lead shield.

**Table 2:** Electron relative stopping and scattering power Pinnacle lookup table

*Physical Density* $\left(g\cdot cm^{-3}\right)$	*Collision Stopping Power Ratio*	*Angular Scattering Power Ratio*
0.000	0.001	0.001
0.291	0.311	0.292
0.927	0.933	0.729
1.000	1.027	0.912
1.047	1.051	1.040
1.100	1.098	1.135
1.427	1.422	1.863
1.940	1.940	3.026
7.000	7.000	9.900

These Pinnacle limitations compound the uncertainty of the heterogeneous dose calculation algorithm resulting in a significant discrepancy between the measured and calculated dose values of up to 25% at the lead edge, with worse agreement in the open field region of the beam for select cases. A trend of increasing disagreement between the calculated and measured dose profiles was observed with increasing electron energy, decreasing distance from the lead edge, and increasing bolus thickness. As a result, it is not recommended that the heterogeneous PBA utilized in the Pinnacle TPS be used to estimate the lens dose in complex treatment situations as those observed here. Instead, reference dose data such as the lens dose profiles presented here should be the primary source of dose estimates, unless a TPS is employed with a properly validated and commissioned heterogeneous electron dose calculation algorithm.

## V. CONCLUSIONS

The dose to the lens of the eye for adjacent facial lesions treated with anterior electron beams can be estimated using the data presented in this study. These measurements provide a useful estimate for the dose that the lens will receive, when shielded with lead, for a clinically‐relevant range of electron energies and bolus thickness combinations. It also allows clinicians to assess the extent of lead margin required to reduce the lens dose to an acceptable level. Furthermore, the dose comparisons between Pinnacle v9.2 and Gafchromic EBT3 film measurements show that the Pinnacle pencil beam electron dose algorithm tends to overestimate the dose deposited under the in‐field lead shield, requiring reference dose data, such as that presented here, to be used in estimating lens doses.

## COPYRIGHT

This work is licensed under a Creative Commons Attribution 4.0 International License.

## Supporting information

Supplementary Material Files

Supplementary Material Files
