# Supplementary figures and images for: Evaluation of lens dose from anterior electron beams: comparison of Pinnacle and Gafchromic EBT3 film
Source: J Appl Clin Med Phys. 2015 Mar 8;17(2):304–14. doi: 10.1120/jacmp.v17i2.5713 (PMC5874807; doi:10.1120/jacmp.v17i2.5713)

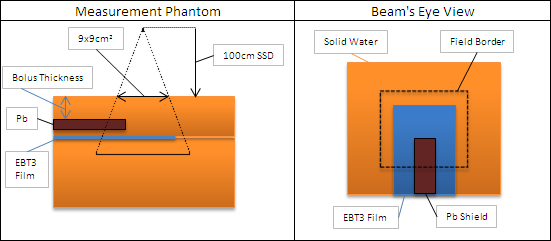

Supplement: Supplementary file 1 — Supplementary Material Files [file ACM2-17-304-s001.png]

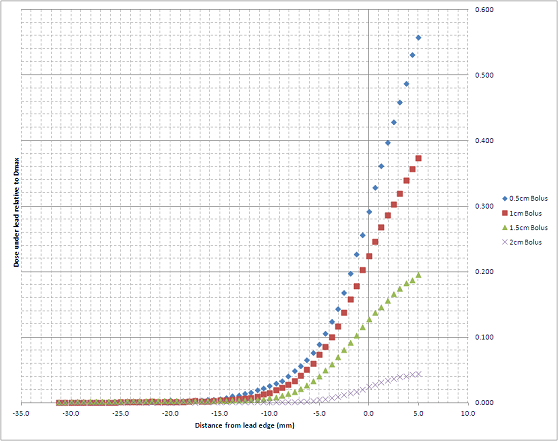

Supplement: Supplementary file 2 — Supplementary Material Files [file ACM2-17-304-s002.png]
